# Incidence of Colon Cancer Among Medicaid Beneficiaries With or Without Human Immunodeficiency Virus Under Comparable Colorectal Cancer Screening Patterns

**DOI:** 10.1093/ofid/ofae246

**Published:** 2024-04-27

**Authors:** Jacqueline E Rudolph, Keri L Calkins, Xueer Zhang, Yiyi Zhou, Filip Pirsl, Xiaoqiang Xu, Eryka Wentz, Bryan Lau, Corinne E Joshu

**Affiliations:** Department of Epidemiology, Bloomberg School of Public Health, Johns Hopkins University, Baltimore, Maryland, USA; Department of Epidemiology, Bloomberg School of Public Health, Johns Hopkins University, Baltimore, Maryland, USA; Mathematica, Ann Arbor, Michigan, USA; Department of Epidemiology, Bloomberg School of Public Health, Johns Hopkins University, Baltimore, Maryland, USA; Department of Epidemiology, Bloomberg School of Public Health, Johns Hopkins University, Baltimore, Maryland, USA; Department of Epidemiology, Bloomberg School of Public Health, Johns Hopkins University, Baltimore, Maryland, USA; Department of Medicine, School of Medicine, Johns Hopkins University, Baltimore, Maryland, USA; Department of Epidemiology, Bloomberg School of Public Health, Johns Hopkins University, Baltimore, Maryland, USA; Department of Epidemiology, Bloomberg School of Public Health, Johns Hopkins University, Baltimore, Maryland, USA; Department of Epidemiology, Bloomberg School of Public Health, Johns Hopkins University, Baltimore, Maryland, USA

**Keywords:** colon cancer, colorectal cancer screening, endoscopy, Medicaid, human immunodeficiency virus

## Abstract

**Background:**

People with human immunodeficiency virus (HIV; PWH) in the United States have a lower incidence of colon cancer than the general population. The lower incidence may be explained by differences in receipt of screening. Thus, we sought to estimate colon cancer incidence under scenarios in which Medicaid beneficiaries, with or without HIV, followed the same screening protocols.

**Methods:**

We used data from 1.5 million Medicaid beneficiaries who were enrolled in 14 US states in 2001–2015 and aged 50–64 years; 72 747 beneficiaries had HIV. We estimated risks of colon cancer and death by age, censoring beneficiaries when they deviated from 3 screening protocols, which were based on Medicaid's coverage policy for endoscopies during the time period, with endoscopy once every 2, 4, or 10 years. We used inverse probability weights to control for baseline and time-varying confounding and informative loss to follow-up. Analyses were performed overall, by sex, and by race/ethnicity.

**Results:**

PWH had a lower incidence of colon cancer than beneficiaries without HIV. Compared with beneficiaries without HIV, the risk difference at age 65 years was −1.6% lower (95% confidence interval, −2.3% to −.7%) among PWH with the 2-year protocol and −0.8% lower (−1.3% to −.3%) with the 10-year protocol. Results were consistent across subgroup and sensitivity analyses.

**Conclusions:**

Our findings suggest that the lower risk of colon cancer that has been observed among PWH aged 50–64 years compared with those without HIV is not due to differences in receipt of lower endoscopy. Keywords: colon cancer, colorectal cancer screening, endoscopy, Medicaid, human immunodeficiency virus

## Teaser

Our findings among 1.5 million Medicaid beneficiaries suggest that the lower risk of colon cancer reported among people with human immunodeficiency virus in the United States compared with the general population is not due to differences in colorectal cancer screening.

In the modern treatment era, the incidence of cancers common in the general population has been increasing among people with human immunodeficiency virus (HIV; PWH) in the United States, partly due to gains in life expectancy [1, 2]. Today, non–AIDS-defining cancers are a leading cause of death among PWH [3–9]. Colorectal cancer (CRC) is one of the most common cancers in the general population [10, 11]. PWH have a similar or lower incidence of CRC than the general population [12–18], despite their having a higher prevalence of risk factors associated with CRC, including social determinants of health like poverty and individual risk factors such as smoking [19].

One proposed mechanism for the lower CRC incidence among PWH is differences in receipt of CRC screening. CRC screening, which before 2021 was recommended to begin at age 50 years, has been an important public health intervention for reducing CRC incidence and mortality rates [20, 21]. This is because the lower endoscopic procedures used for screening (eg, colonoscopy) can detect cancer early and also prevent cancer through the removal of precancerous polyps [22]. Thus, if PWH and those without HIV undergo lower endoscopic procedures at different rates, this could affect their lifetime risk of colon cancer. Prior studies comparing CRC screening by HIV status have generally reported a similar or lower rates of screening among PWH [18, 23–26].

We previously explored colon cancer incidence and lower endoscopy among Medicaid beneficiaries with or without HIV [27, 28]. Medicaid is a joint federal and state insurance program that provides coverage to individuals meeting certain eligibility criteria, primarily related to low income, and is the largest single source of insurance for PWH in the United States [29]. Among Medicaid beneficiaries aged 50–64 years, we reported a lower incidence of colon cancer among PWH than among beneficiaries without HIV but a similar or higher incidence of endoscopy [28]. In the current study, we sought to estimate colon cancer incidence under scenarios where Medicaid beneficiaries aged 50–64 years, with or without HIV, followed the same CRC screening protocols. Understanding whether differences in colon cancer incidence between PWH and people without HIV is due to differences in screening uptake is critical for informing high-quality care for aging PWH.

## METHODS

### Study Sample

We drew on Medicaid Analytic eXtract and Transformed Medicaid Statistical Information System Analytic Files data for beneficiaries who enrolled in 2001–2015 from 14 US states: Alabama, California, Colorado, Florida, Georgia, Illinois, Massachusetts, Maryland, North Carolina, New York, Ohio, Pennsylvania, Texas, and Washington. For all beneficiaries, we identified the first continuous period of enrollment (hereafter, first eligibility period) in Medicaid that was >6 months in length. During this time period, average-risk adults began CRC screening at age 50 years; thus, we included beneficiaries aged 50–64 years during their first eligibility period.

To ensure that we did not miss healthcare encounters not covered by Medicaid, we excluded beneficiaries without full benefits, those with dual enrollment in Medicare or private insurance, and those aged ≥65 years (due to Medicare eligibility). The analytic baseline was defined as the date when a beneficiary turned 50 years old or 6 months after the start of the first eligibility period, whichever occurred last. We excluded beneficiaries who had any cancer-related claim before baseline or a lower endoscopy before age 50 years, as they may have had a polyp removed and would not have a colon cancer risk comparable to that of beneficiaries who had not undergone endoscopy.

This study was approved by the Johns Hopkins Bloomberg School of Public Health Institutional Review Board. It represents a secondary analysis of administrative claims data, and informed consent was not required.

### Measures

All measures were defined using *International Classification of Diseases, Ninth Revision* (*ICD-9*) and/or Current Procedural Terminology/Healthcare Common Procedure Coding System codes on Medicaid inpatient or outpatient claims (see Supplementary Table 1 for more details). HIV and colon cancer diagnoses required 1 inpatient or 2 outpatient claims within 2 years with a relevant code; all other variables required 1 claim.

Our first exposure was HIV status at baseline. For computational efficiency, we used a 25% random sample of beneficiaries without HIV as controls. Our second exposure was time-updated receipt of colonoscopy or sigmoidoscopy (hereafter, endoscopy). In claims data, it is difficult to distinguish screening from diagnostic endoscopy [28]. Thus, we defined screening endoscopy as either the first endoscopy observed or any subsequent endoscopy that occurred >1 year since the last observed endoscopy. Endoscopies not meeting this definition were excluded, as multiple endoscopies within a short time period are likely to be for diagnostic purposes or for follow-up to a positive screening endoscopy.

Our outcome was incidence of colon cancer as the first primary cancer. We restricted to colon cancer because, as has been reported elsewhere, there was potential for misclassification of anal cancers as rectal cancers differentially by HIV status, made even more likely in our data under *ICD-9* coding [30]. To further reduce potential misclassification, we considered colon cancer to be anal cancer if an anal cancer diagnosis occurred within 90 days of the colon cancer diagnosis.

We used the personal summary file to determine race/ethnicity (non-Hispanic white, non-Hispanic Back, Hispanic, or other), sex (male or female), state, and calendar period (2001–2005, 2006–2010, or 2011–2015) at baseline. We identified Charlson comorbidities (excluding HIV and cancer) and calculated the time-updated number of comorbid conditions (categorized as 0, 1, or ≥2 conditions), as a marker of multimorbidity burden and healthcare needs.

### Statistical Analysis

We followed beneficiaries from baseline until the first of the following events: (1) colon cancer diagnosis, (2) other cancer diagnosis, (3) death, (4) loss of Medicaid eligibility, or (5) end of ICD-9 coding (30 September 2015). To estimate colon cancer incidence, we used discrete time survival analysis, with time binned into 3-month intervals. In each interval, we assessed whether a beneficiary had the outcome, died, or was censored due to loss of eligibility or occurrence of another cancer type. We treated death as a competing event.

We also assessed whether a beneficiary remained adherent to the screening protocol during each 3-month interval. During the study period, Medicaid provided coverage for individuals aged ≥50 years to undergo colonoscopy every 2 years if high risk (eg, family history of CRC) or every 10 years if average risk or sigmoidoscopy every 4 years [31]. Based on this coverage, we defined 2 screening protocols of interest, requiring participants to undergo endoscopy once every 2 years or once every 10 years of follow-up. We allowed a bandwidth of 3 months around both of these definitions, such that under the 2-year protocol an endoscopy could be received every 21–27 months. Participants were censored if they were observed for longer than the specified time period without endoscopy or if the time between endoscopies was less than this period.

We estimated the cumulative incidence (hereafter, risk) functions by HIV status for colon cancer and death, using age as the time scale [32]. By using age as the time scale, we assumed that the follow-up observed among individuals at younger ages (say, 50–51 years) represented what was seen at those ages among beneficiaries we observed at older ages (say, 60–61 years). We estimated risk functions with or without weighting for baseline HIV status, time-varying adherence to the screening protocol, and time-varying loss of Medicaid enrollment (see Appendix 1 and Supplementary Table 2 for details). These weights were used to control for confounders of the relationship between HIV status and colon cancer (race/ethnicity, sex, state, and calendar period), confounders of the relationship between CRC screening and colon cancer (all baseline confounders plus time-varying number of comorbid conditions), and variables related to nonrandom right censoring and the outcome (same variables as for screening). The weighted risk functions by HIV status estimate incidence of colon cancer under 2 scenarios: (1) all beneficiaries had HIV, always followed the screening protocol, and were not censored or (2) all beneficiaries did not have HIV, always followed the screening protocol, and were not censored. We compared risk by HIV status to estimate risk difference (RD) and risk ratio functions.

We carried out analyses overall and by sex and race. We repeated our analysis subset to the 2010–2015 calendar period, as this period aligns with a more modern era of ART therapy and changes to CRC screening practice (eg, a shift away from sigmoidoscopy). We also conducted 3 sensitivity analyses. First, we considered only colonoscopies as screening procedures. Second, we implemented a screening protocol requiring participants to undergo endoscopy once every 4 years. Third, we allowed screening endoscopies to occur within 6 months of each other. All analyses were carried out using R software, version 4.0.5 (R Foundation). Code for this analysis is available on GitHub (https://github.com/jerudolph13/medican_crc_screening).

## RESULTS

Our analysis included 1 528 414 beneficiaries, who contributed 40 428 065 person-months of follow-up and had a median follow-up of 14.7 months (interquartile range, 6.0–32.0 months). There were 72 747 beneficiaries with HIV, who contributed 2 632 999 person-months of follow-up and had a median follow-up of 20.0 months (interquartile range, 9.0–53.0 months). At baseline (Table 1), beneficiaries with HIV were younger than those without HIV, with median ages of 51.9 and 56.1 years, respectively, perhaps because 38% of beneficiaries with HIV were enrolled before age 50 years, compared with 15% of beneficiaries without HIV. Beneficiaries with HIV were more likely to be non-Hispanic Black and male and had more chronic conditions (Table 1).

**Table 1.** Characteristics of Medicaid Beneficiaries at Baseline, Overall, and by Human Immunodeficiency Virus Status

Characteristic	Medicaid Beneficiaries, No. (%)^a^		
	Overall (n = 1 528 414)	With HIV (n = 72 747)	Without HIV (n = 1 455 667)
Age, median (IQR), y	55.9 (51.5–60.9)	51.9 (50.0–57.6)	56.1 (51.7–61.0)
Sex			
Female	815 759 (53.4)	25 586 (35.2)	790 173 (54.3)
Male	712 627 (46.6)	47 160 (64.8)	665 467 (45.7)
Unknown	28 (0.0)	1 (0.0)	27 (0.0)
Race/ethnicity			
Non-Hispanic white	627 385 (41.0)	16 990 (23.4)	610 395 (41.9)
Non-Hispanic Black	320 472 (21.0)	35 111 (48.3)	285 361 (19.6)
Hispanic	210 418 (13.8)	6001 (8.2)	204 417 (14.0)
Other/unknown^b^	370 139 (24.2)	14 645 (20.1)	355 494 (24.4)
Enrolled before age 50 y	904 750 (15.3)	28 311 (38.3)	876 439 (15.0)
Enrollment period			
2001–2005	450 025 (29.4)	22969 (31.6)	427056 (29.3)
2006–2010	314 879 (20.6)	18154 (25.0)	296725 (20.4)
2011–2015	763 510 (50.0)	31624 (43.5)	731886 (50.3)
State			
Alabama	24 768 (1.6)	695 (1.0)	24 073 (1.7)
California	476 300 (31.2)	11 658 (16.0)	464 642 (31.9)
Colorado	14 572 (1.0)	285 (0.4)	14 287 (1.0)
Florida	83 794 (5.5)	7814 (10.7)	75 980 (5.2)
Georgia	44 745 (2.9)	2712 (3.7)	42 033 (2.9)
Illinois	111 170 (7.3)	3612 (5.0)	107 558 (7.4)
Massachusetts	64 189 (4.2)	3276 (4.5)	60 913 (4.2)
Maryland	36 406 (2.4)	2533 (3.5)	33 873 (2.3)
North Carolina	50 353 (3.3)	2427 (3.3)	47 926 (3.3)
New York	290 905 (19.0)	29 655 (40.8)	261 250 (17.9)
Ohio	79 630 (5.2)	1396 (1.9)	78 234 (5.4)
Pennsylvania	94 684 (6.2)	2090 (2.9)	92 594 (6.4)
Texas	90 459 (5.9)	3387 (4.7)	87 072 (6.0)
Washington	66 439 (4.3)	1207 (1.7)	65 232 (4.5)
No. of comorbid conditions			
0	881 285 (57.7)	28 568 (39.3)	852 717 (58.6)
1	355 077 (23.2)	20 938 (28.8)	334 139 (23.0)
≥2	292 052 (19.1)	23 241 (31.9)	268 811 (18.5)

Abbreviations: HIV, human immunodeficiency virus; IQR, interquartile range.

^a^Data represent no. (%) of beneficiaries unless otherwise specified.

^b^Other includes anyone classified as American Indian or Alaska native, Asian, Native Hawaiian or other Pacific Islander, or >1 race, without indication of Hispanic ethnicity.

We observed 3157 diagnoses of colon cancer and 27 301 deaths during follow-up. The crude risk of colon cancer among beneficiaries with HIV was 0.3% (95% confidence interval [CI], 0.2%–.4%) by age 55, 0.6% (0.5%–0.7%) by age 60, and 1.1% (9.0%­­–1.3%) by age 65 years (Supplementary Table 3). The respective risks for beneficiaries without HIV were marginally higher, resulting in RDs at ages 55, 60, and 65 years of −0.1% (95% CI, −0.2% to −0.1%), −0.3% (−0.4% to −0.2%), and −0.3% (−0.5% to 0.1%), respectively. When we controlled for baseline HIV status and loss to follow-up (Supplementary Table 3), the estimated risks were slightly larger and RDs were stronger. The RDs at ages 55, 60, and 65 years were −0.3% (95% CI, −0.5% to −0.2%), −0.6% (−1.0% to −0.2%), and −0.7% (−1.2% to 0.1%), respectively.

**Figure 1.**
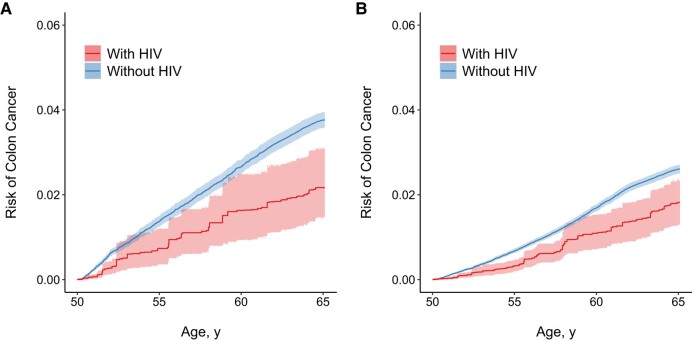
Risk of colon cancer from age 50 to 64 years by human immunodeficiency virus (HIV) status, with weighting for baseline HIV status and for screening protocols requiring endoscopy once every 2 (*A*) or 10 (*B*) years. Solid lines represent risks of colon cancer by age; shaded areas represent 95% confidence intervals for risks.

Under the 2-year screening protocol, we saw markedly higher estimates of colon cancer risk among beneficiaries with or without HIV (Table 2 and Figure 1*A*), but risks remained lower at all ages among beneficiaries with HIV. Among beneficiaries with HIV, colon cancer risk was 0.9% (95% CI, 0.5­%–1.5%) by age 55, 1.7% (1.0%­–2.5%) by age 60, and 2.2% (1.5%– 3.0%) by age 65 years. The RDs were also stronger relative to the crude and HIV-weighted analyses: −0.7% (95% CI, −1.2% to −0.2%) at age 55, −1.3% (−1.9% to −0.4%) at age 60, and −1.6% (−2.3% to −0.7%) at age 65 years. Under the 10-year screening protocol, risk estimates were lower (Table 2 and Figure 1*B*). Among beneficiaries with HIV, the colon cancer risk was 0.5% (95% CI, 0.3%–0.7%) at age 55, 1.3% (0.9%–1.7%) at age 60, and 1.8% (1.3%–2.3%) by age 65 years. The RDs at ages 60 and 65 years were also weaker: −0.4% (95% CI, −0.6% to −0.2%) at age 55, −0.7% (−1.1% to −0.2%) at age 60, and −0.8% (−1.3% to −0.3%) at age 65 years.

**Table 2.** Risk of Colon Cancer and Risk Contrasts by Beneficiary Age, Sex, Human Immunodeficiency Virus Status, and Screening Protocol

Sex and Age Group	Screening Every 2 y				Screening Every 10 y			
	Risk With HIV (%)	Risk Without HIV (%)	RD (%)	RR	Risk With HIV (%)	Risk Without HIV (%)	RD (%)	RR
All beneficiaries by age, y								
55	0.9	1.7	−0.7 (−1.2 to −0.2)	0.57 (0.33–0.88)	0.5	0.9	−0.4 (−0.6 to −0.2)	0.57 (0.37–0.82)
60	1.7	2.9	−1.3 (−1.9 to −0.4)	0.57 (0.34–0.86)	1.3	1.9	−0.7 (−1.1 to −0.2)	0.65 (0.45–0.88)
65	2.2	3.8	−1.6 (−2.3 to −0.7)	0.58 (0.39–0.80)	1.8	2.6	−0.8 (−1.3 to −0.3)	0.7 (0.51–0.89)
Female beneficiaries by age, y								
55	0.8	1.8	−1.0 (−1.6 to −0.2)	0.43 (0.15–0.87)	0.4	0.8	−0.4 (−0.6 to −0.1)	0.50 (0.25–0.82)
60	1.2	3.2	−2.0 (−2.8 to −1.1)	0.36 (0.15–0.66)	1.2	2.0	−0.8 (−1.4 to 0.0)	0.61 (.32, 1.02)
65	1.9	3.9	−2.0 (−2.9 to −0.7)	0.49 (0.26–0.82)	1.8	2.5	−0.7 (−1.4 to 0.2)	0.72 (0.44– 1.07)
Male beneficiaries by age, y								
55	0.9	1.5	−0.6 (−1.1 to 0.0)	0.61 (0.30–1.00)	0.5	0.9	−0.4 (−0.6 to −0.1)	0.53 (0.27–0.92)
60	1.9	3.0	−1.1 (−2.0 to 0.0)	0.64 (0.35–1.01)	1.2	2.0	−0.8 (−1.3 to −0.3)	0.59 (0.37–0.86)
65	2.2	3.9	−1.8 (−2.6 to −0.6)	0.55 (0.35–0.85)	1.7	2.8	−1.1 (−1.6 to −0.4)	0.60 (0.41–0.86)

Abbreviations: HIV, human immunodeficiency virus; RD, risk difference; RR, risk ratio.

Findings were largely similar among female and male beneficiaries (Table 2 and Supplementary Figures 1–2). Colon cancer risk was lowest among Hispanic beneficiaries regardless of HIV status and, among beneficiaries without HIV, highest among non-Hispanic black beneficiaries (Supplementary Table 4 and Supplementary Figures 3-5). We again estimated that colon cancer risk was lower among beneficiaries with HIV than among those without HIV, regardless of race/ethnicity group.

**Figure 2.**
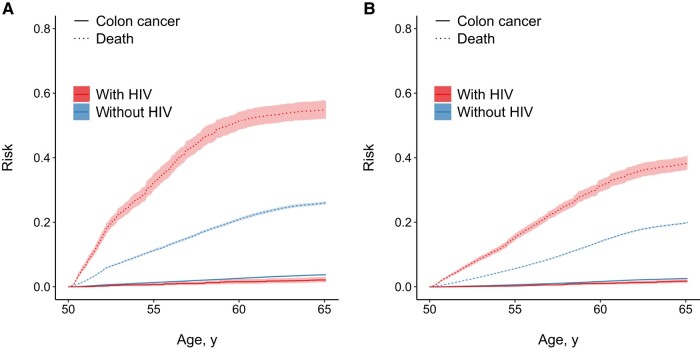
Risks of colon cancer and death from age 50 to 64 years by human immunodeficiency virus (HIV) status, weighting for baseline HIV status and for screening protocols requiring endoscopy once every 2 (*A*) or 10 (*B*) years. Solid lines represent risks of colon cancer by age; dotted lines, risks of death by age; shaded areas, 95% confidence intervals for risks.

The risk of death was higher among beneficiaries with HIV in all analyses (Supplementary Table 5 and Figure 2). As for colon cancer, the risk of death was lower and RDs weaker under the 10-year relative to the 2-year protocol. In general, the risk of death was higher at all ages among male beneficiaries without HIV relative to female beneficiaries without HIV, resulting in weaker RDs across HIV status among male beneficiaries (Supplementary Table 5). When stratifying by race/ethnicity (Supplementary Table 6), non-Hispanic black beneficiaries with HIV had the highest risk of death by age 65 years, resulting in stronger RDs relative to non-Hispanic black beneficiaries without HIV. The mortality rate disparity was particularly noticeable under the 2-year protocol.

When we subset the analysis to 2010–2015 (Supplementary Table 7), the colon cancer risk was marginally lower for beneficiaries with or without HIV, across all protocols. For example, the colon cancer risk by age 65 years was 1.5% (95% CI, 1.0%–2.0%) and 2.2% (2.1%–2.3%) among beneficiaries with or without HIV, respectively. RDs were attenuated under the 2-year protocol but were similar under the 10-year protocol. In the sensitivity analyses (Supplementary Table 7), the results were nearly identical to those of the main analysis. Under the 4-year screening protocol, risk and RD estimates were similar to those with the 2-year protocol (Supplementary Tables 7 and 8).

## DISCUSSION

Here, we assessed lower endoscopy and colon cancer incidence among 1.5 million Medicaid beneficiaries aged 50–64 years—of whom >72 000 had HIV—from 14 states over 15 years. We saw that the incidence of colon cancer would have been higher among beneficiaries without HIV than among comparable beneficiaries with HIV had all beneficiaries followed a similar CRC screening protocol. Our results were consistent by sex and race/ethnicity. These data suggest that the lower risk of colon cancer that has been observed among PWH compared with those without HIV is not due to differences in receipt of lower endoscopy between 50 and 64 years of age.

We also observed that the risk of colon cancer (and of death) was highest regardless of HIV status under a protocol specifying that participants undergo endoscopy every 2 years. This finding likely reflects the recommendation that “high-risk” individuals receive screening at this rate [31]. With more years allowed between endoscopies, the colon cancer risk dropped in beneficiaries with and without HIV, and the RD between the 2 groups was attenuated. Results were also attenuated when the data were subset to the 2010–2015 period.

Across all analyses, beneficiaries with HIV had a lower incidence of colon cancer than those without HIV. Our findings agree with those of a previous study that indirectly controlled for screening by examining colon cancer separately by stage at diagnosis and found that colon cancer rates were lower among PWH (relative to the general US population) regardless of cancer stage or tumor size [15]. This suggests that there is a mechanism beyond receipt of recommended screening causing the deficit in cancer diagnoses. For example, our prior work in Medicaid showed that PWH under 50 years of age are more likely to undergo lower endoscopy than beneficiaries without HIV [28]. PWH may thus be more likely to have precancerous polyps removed early, lowering their risk of cancer after age 50 years.

It is well established that screening endoscopy reduces the risk of colon cancer diagnosis and death [20, 33]. Generally, prior studies have compared outcomes between scenarios where screening is received and those where screening is not received, which differs from the comparison made in our analysis. We were unable to generate the scenario where participants were not undergoing endoscopy, due to how colon cancer diagnoses are identified in claims data. Moreover, it is possible that the Medicaid population is distinct from those previously studied. Individuals insured by Medicaid who are seeking CRC screening may have a higher burden of CRC risk factors than those who do not seek screening. We have no measure of CRC family history or CRC lifestyle risk factors (eg, diet, physical activity, and smoking), so we cannot assess whether individuals who remained adherent to the protocol had a higher prevalence of these risk factors. It is also possible that we are missing the relevant age window for risk reduction because we censor individuals when they become eligible for Medicare at 65 years. It has been suggested that the largest reductions in CRC risk due to screening would be observed among older adults [20].

Furthermore, we cannot rule out that our results are due to the impact of deaths among PWH. We saw that the risk of death by age 65 years among beneficiaries with HIV was more than twice that among beneficiaries without HIV. This may reflect the time period under study; most of our data preceded the recommendation in 2012 to treat all PWH regardless of CD4 cell count, although findings were similar when subset to 2010–2015. While others have reported a decreased mortality gap between PWH and the general US population by 2015 [2], it is possible that PWH on Medicaid had higher mortality rates than other PWH during this period [34]. Previous work has shown that PWH on Medicaid are less likely to be retained in HIV care and virally suppressed than PWH on private insurance or Medicare [35].

Several limitations of our analysis merit discussion. First, some possible confounders of the relationship between screening and colon cancer incidence cannot be measured in Medicaid, particularly lifestyle risk factors. Second, while we controlled for variables related to disenrollment from Medicaid [36, 37], we were unable to ascertain why individuals disenrolled (eg, whether disenrollment was due to incomplete paperwork or higher income). Different disenrollment mechanisms could have different relationships with the outcome; ideally, we would build separate weights for each. Third, as mentioned above, our results apply only to individuals aged 50–64 years; in the general population, much of the colon cancer burden occurs among individuals aged ≥65 years. That said, approximately 87% of PWH aged ≥50 years were aged 50–64 years at the end of our study period [38].

Fourth, we lack historical data on beneficiaries; thus, it is possible that we unintentionally included some who underwent endoscopy before age 50 years with removal of precancerous polyps, reducing their risk at older ages. This may occur differentially among PWH because, as noted previously, we have observed a higher endoscopy rate under age 50 years in this group. Fifth, we evaluated screening protocols that aligned with Medicaid coverage during the study time period [31]. We note that these screening protocols do not perfectly align with those recommended by gastroenterological societies and thus some private insurance coverage during this time period, although there is substantial overlap. [39]. Finally, we assumed that all of a beneficiary's healthcare encounters were captured in claims data for the duration of their Medicaid enrollment. While this was reasonable given our exclusion criteria (eg, of individuals dually enrolled or with restricted benefits), the assumption would be violated if the state had incomplete reporting in a given year. Medicaid claims are known to have data quality issues; unfortunately, there was not routine reporting of data quality during the period of this study, as there is for more recent Medicaid data [40].

In this study of 1.5 million Medicaid beneficiaries 50–64 years old, we estimated that PWH would have a lower incidence of colon cancer than beneficiaries without HIV even if all beneficiaries followed a similar CRC screening protocol. Under the 10-year screening protocol, there was an approximately 1% difference in incidence by age 65 years. Applied to the almost 600 000 PWH in the United States currently aged ≥50 years, this finding translates to roughly 6000 fewer colon cancer cases by age 65 years among PWH than among individuals without HIV [41]. Outside of simulation studies, few analyses have examined how colon cancer incidence changes under different cancer screening protocols; [33, 42] to our knowledge, no study comparing colon cancer incidence between PWH and people without HIV has controlled for time-varying screening patterns. Our findings suggest that differences in colon cancer incidence between the ages of 50 and 64 years by HIV status were not due to differences in CRC screening. Future studies are needed to examine other possible mechanisms driving the difference in colon cancer incidence, such as biological mechanisms or receipt of lower endoscopy at younger ages.

## Supplementary Material

ofae246_Supplementary_Data

## Supplementary Data

Supplementary materials are available at *Open Forum Infectious Diseases* online. Consisting of data provided by the authors to benefit the reader, the posted materials are not copyedited and are the sole responsibility of the authors, so questions or comments should be addressed to the corresponding author.
