# Supplementary material for: Incidence of Colon Cancer Among Medicaid Beneficiaries With or Without Human Immunodeficiency Virus Under Comparable Colorectal Cancer Screening Patterns
Source: Open Forum Infect Dis. 2024 Apr 27;11(5):ofae246. doi: 10.1093/ofid/ofae246 (PMC11127480; doi:10.1093/ofid/ofae246)
Supplement: ofae246_Supplementary_Data [file ofae246_supplementary_data.docx]

**Appendix Table of Contents.**

| Item | Description | Page |
| --- | --- | --- |
| Appendix 1 | Details on methods | 2 |
| Table S1 | Codes used to define measures | 4 |
| Table S2 | Distribution of final weights summarized across ages, for each screening protocol definition | 6 |
| Table S3 | Risk of colon cancer and risk contrasts by HIV status, for the crude and HIV-weighted analyses | 7 |
| Figure S1 | Risk of colon cancer by HIV status among female beneficiaries, weighting for baseline HIV status and for following screening protocols requiring one endoscopy every (A) two and (B) ten years | 8 |
| Figure S2 | Risk of colon cancer by HIV status among male beneficiaries, weighting for baseline HIV status and for following screening protocols requiring one endoscopy every (A) two and (B) ten years | 9 |
| Table S4 | Risk of colon cancer and risk contrasts by HIV status and race/ethnicity, for each screening protocol definition | 10 |
| Figure S3 | Risk of colon cancer by HIV status among non-Hispanic Black beneficiaries, weighting for baseline HIV status and for following screening protocols requiring one endoscopy every (A) two and (B) ten years | 11 |
| Figure S4 | Risk of colon cancer by HIV status among non-Hispanic White beneficiaries, weighting for baseline HIV status and for following screening protocols requiring one endoscopy every (A) two and (B) ten years | 12 |
| Figure S5 | Risk of colon cancer by HIV status among Hispanic beneficiaries, weighting for baseline HIV status and for following screening protocols requiring one endoscopy every (A) two and (B) ten years | 13 |
| Table S5 | Risk (%) of death and risk contrasts by HIV status, for each screening protocol definition | 14 |
| Table S6 | Risk (%) of death and risk contrasts by HIV status and race/ethnicity, for each screening protocol definition | 15 |
| Table S7 | Risk (%) of colon cancer and risk contrasts by HIV status, under the sensitivity analyses | 16 |
| Table S8 | Risk (%) of colon cancer, death, and risk contrasts by HIV status, for the 4-year screening protocol definition | 17 |

**Appendix 1. Details on methods**

To control for confounding by baseline HIV status, we built stabilized inverse probability of treatment weights:

$w_{h}=\hat{P}(H=h)/\hat{P}(H=h|\mathbf{X,}A_{1})$,

where $H=h$ represents a beneficiary’s observed HIV status and $\mathbf{X}$ represents the set of baseline confounders, including race/ethnicity, sex, state, and calendar period. We additionally included baseline age ($A_{1}$), specified using cubic splines. The exposure probabilities were estimated using logistic regression.

To control for time-varying confounders of the relationship between receipt of endoscopy and colon cancer incidence, we used time-varying inverse probability of censoring weights defined as:

$$w_{s}\left( t \right)=\prod_{k=1}^{t} \frac{I\left( S_{k}=1 \right) \hat{P}(S_{k}=1|H,{A_{t},S}_{k-1},S_{k-2})}{\hat{P}(S_{k}=1|H,\mathbf{X},A_{t},S_{k-1},S_{k-2},D_{k},D_{k-1})}$$

where $S_{t}=1$ represents following the screening protocol at time interval $t$ and $D_{t}$ represents a beneficiary’s time-varying number of comorbidities. We estimated the numerator and denominator screening probabilities using pooled logistic regression, with models that were stratified by baseline HIV status.

Finally, we estimated inverse probability of censoring weights to control for differential right censoring (due to loss of Medicaid eligibility or occurrence of other cancers):

$$w_{c}\left( t \right)=\prod_{k=1}^{t} \frac{I\left( C_{k}=1 \right) \hat{P}(C_{k}=1|H,A_{t},S_{k})}{\hat{P}(C_{k}=1|H,\mathbf{X},A_{t},S_{k},S_{k-1},S_{k-2},D_{k},D_{k-1})}$$

where $C_{t}=1$ indicates that the beneficiary had not yet been censored at time $t$. For beneficiaries who were right censored, we created an artificial record, with 1 day of follow-up time, which was the record in which they were censored (i.e., their weight was equal to zero). We estimated the numerator and denominator censoring probabilities using pooled logistic regression. The final weights in any given period were defined as:

$$w\left( t \right)=w_{h}\times w_{s}\left( t \right)\times w_{c}(t)$$

Across the screening protocol analyses, the distributions of cumulative weights were well behaved, suggesting no practical violations of the positivity assumption (Table S2).

Under the assumptions of exchangeability, causal consistency, and positivity, we can interpret the RD (or RR) function as a contrast of counterfactual scenarios across ages:

$$RD\left( t \right)=E\left[ Y\left( t \right)^{h=1,\bar{s}=\bar{1},\bar{c}=\bar{1}} \right]-E[Y\left( t \right)^{h=0,\bar{s}=\bar{1},\bar{c}=\bar{1}}]$$

where $E\left[ Y\left( t \right) \right]$ is risk of colon cancer through time $t$. We use superscripts to denote counterfactual outcomes under interventions on $H$, $S$, and $C$. Overbars represent history of a variable through time $t$. For example, the intervention $\bar{s}=\bar{1}$ implies setting someone to follow the screening protocol at all time points $j\leq t$.

We also estimated and contrasted risk functions under interventions on baseline HIV status and right censoring:

$$RD\left( t \right)=E\left[ Y\left( t \right)^{h=1,\bar{c}=\bar{1}} \right]-E[Y\left( t \right)^{h=0,\bar{c}=\bar{1}}]$$

Note that these risks have interventions on $H$ and $C$ but not $S$.

To estimate 95% confidence intervals around the weighted risk functions, we used the 2.5^th^ and 97.5^th^ percentiles of risk point estimates from 500 bootstrap resamples. For the crude risk function, we used the extension of Greenwood’s formula.

Table S1. Codes^a^ used to define measures

| Variable | CPT/HCPCS | ICD-9 |
| --- | --- | --- |
| Human Immunodeficiency Virus^b^ | --- | 042-044, 079.53, 795.71, V08 |
| Colon Cancer | --- | 153.X |
| Colonoscopy^c^ | 45355, 45378, 45379, 45380, 45381, 45382, 45383, 45384, 45385, 45386, 45387, 45388, 45389, 45390, 45391, 45392, 45393, 45398, G0105, G0121 | 45.23, 45.25, 45.42, 76.51 |
| Sigmoidoscopy^c^ | 45330, 45331, 45332, 45333, 45334, 45335, 45337, 45338, 45339, 45340, 45341, 45342, 45343, 45344, 45345, 45346, 45347, 45349, 45350, G0104 | 45.24 |
| Charlson comorbidities^d^ |  |  |
| Myocardial infarction | --- | 410.X, 412.X |
| Congestive heart failure | --- | 398.91, 402.01, 402.11, 402.91, 404.01, 404.03, 404.11, 404.13, 404.91, 404.93, 425.4-425.9, 428.X |
| Peripheral vascular disease | --- | 093.0, 437.3, 440.X, 441.X, 443.1-443.9, 447.1, 557.1, 557.9, V43.4 |
| Cerebrovascular disease | --- | 362.34, 430.X-438.X |
| Dementia | --- | 290.X, 294.1, 331.2 |
| Chronic pulmonary disease | --- | 416.8, 416.9, 490.X-505.X, 506.4, 508.1, 508.8 |
| Rheumatic disease | --- | 446.5, 710.0-710.4, 714.0-714.2, 714.8, 725.X |
| Peptic ulcer disease | --- | 531.X-534.X |
| Mild liver disease | --- | 070.22, 070.23, 070.32, 070.33, 070.44, 070.54, 070.6, 070.9, 570.X, 571.X, 573.3, 573.4, 573.8, 573.9, V42.7 |
| Moderate/severe liver disease | --- | 456.0-456.2, 572.2-572.8 |
| Diabetes without chronic complication | --- | 250.0-250.3, 250.8, 250.9 |
| Diabetes with chronic complication | --- | 250.4-250.7 |
| Hemiplegia or paraplegia | --- | 334.1, 342.X, 343.X, 344.0-344.6, 344.9 |
| Renal disease | --- | 403.01, 403.11, 403.91, 404.02, 404.03, 404.12, 404.13, 404.92, 404.93, 582.X, 583.0-583.7, 585.X, 586.X, 588.0, V42.0, V45.1, V56.X |
| Abbreviations: CPT, current procedural terminology; HCPCS, healthcare common procedure coding system; ICD, international classification of diseases  ^a^A code ending with “.X” indicates a wildcard. Any number(s) could appear after the decimal place.  ^b^Definition based on https://www2.ccwdata.org/web/guest/condition-categories-other  ^c^Definition based on doi:10.1016/j.amepre.2019.04.010; claim included as long as it did not have a procedure modifier code of 52, 53, 73, or 74  ^d^Definition based on doi:10.1093/aje/kwq433 | | |

Table S2. Distribution of final weights from the censoring weight analysis summarized across ages, for each screening protocol definition

| Analysis | Mean | Minimum | Maximum |
| --- | --- | --- | --- |
| 2 Years |  |  |  |
| Mean weight | 0.92 | 0.37 | 1.26 |
| Minimum weight | <0.01 | 0.00 | 0.86 |
| Maximum weight | 5.03 | 1.19 | 28.40 |
| 4 Years |  |  |  |
| Mean weight | 0.92 | 0.33 | 1.13 |
| Minimum weight | <0.01 | 0.00 | 0.74 |
| Maximum weight | 5.85 | 1.20 | 38.10 |
| 10 Years |  |  |  |
| Mean weight | 0.89 | 0.31 | 1.05 |
| Minimum weight | <0.01 | 0.00 | 0.50 |
| Maximum weight | 6.87 | 1.20 | 67.30 |

Table S3. Risk (%) of colon cancer and risk contrasts by HIV status, for the crude and HIV-weighted analyses

| Age | Crude | | | | HIV Weights | | | |
| --- | --- | --- | --- | --- | --- | --- | --- | --- |
|  | With HIV | Without HIV | RD | RR | With HIV | Without HIV | RD | RR |
| 55 | 0.3 | 0.4 | -0.1  (-0.2, -0.1) | 0.65  (0.48, 0.83) | 0.5 | 0.8 | -0.3  (-0.5, -0.2) | 0.58  (0.38, 0.81) |
| 60 | 0.6 | 0.9 | -0.3  (-0.4, -0.2) | 0.67  (0.53, 0.81) | 1.1 | 1.8 | -0.6  (-1.0, -0.2) | 0.64  (0.44, 0.88) |
| 65 | 1.1 | 1.4 | -0.3  (-0.5, -0.1) | 0.81  (0.66, 0.96) | 1.8 | 2.5 | -0.7  (-1.2, -0.1) | 0.73  (0.52, 0.94) |


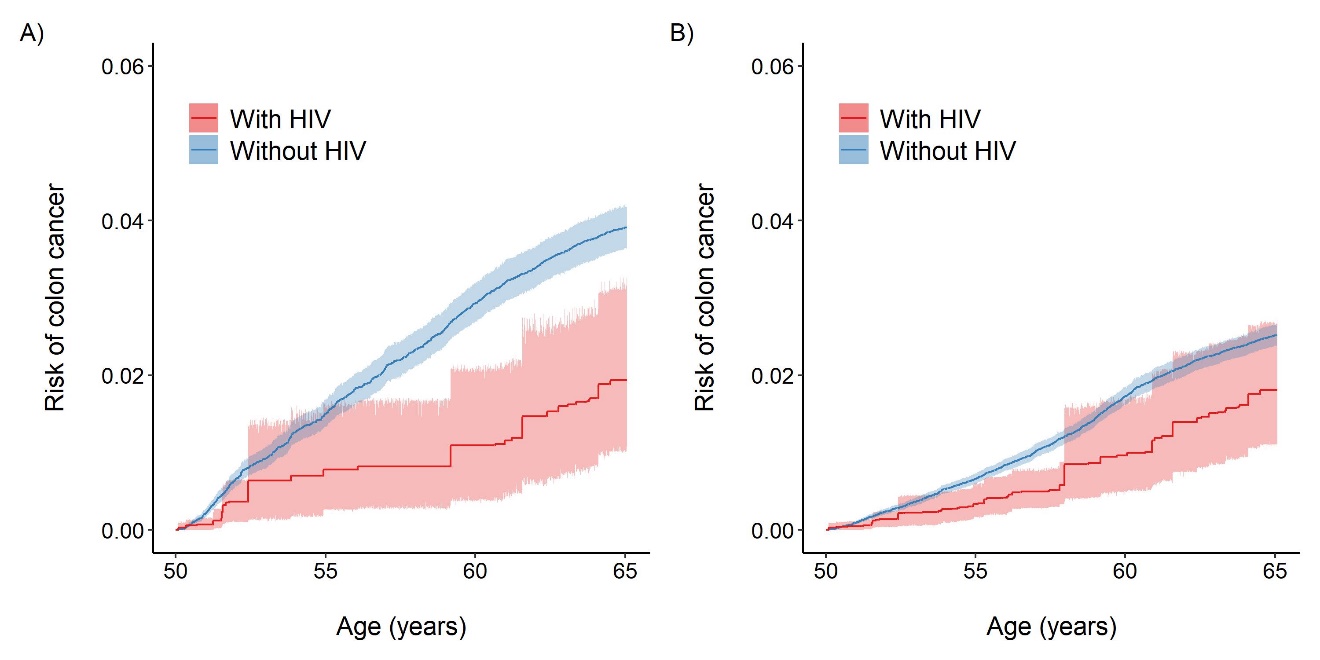


Figure S1. Risk of colon cancer by HIV status among female beneficiaries, weighting for baseline HIV status and for following screening protocols requiring one endoscopy every (A) two and (B) ten years


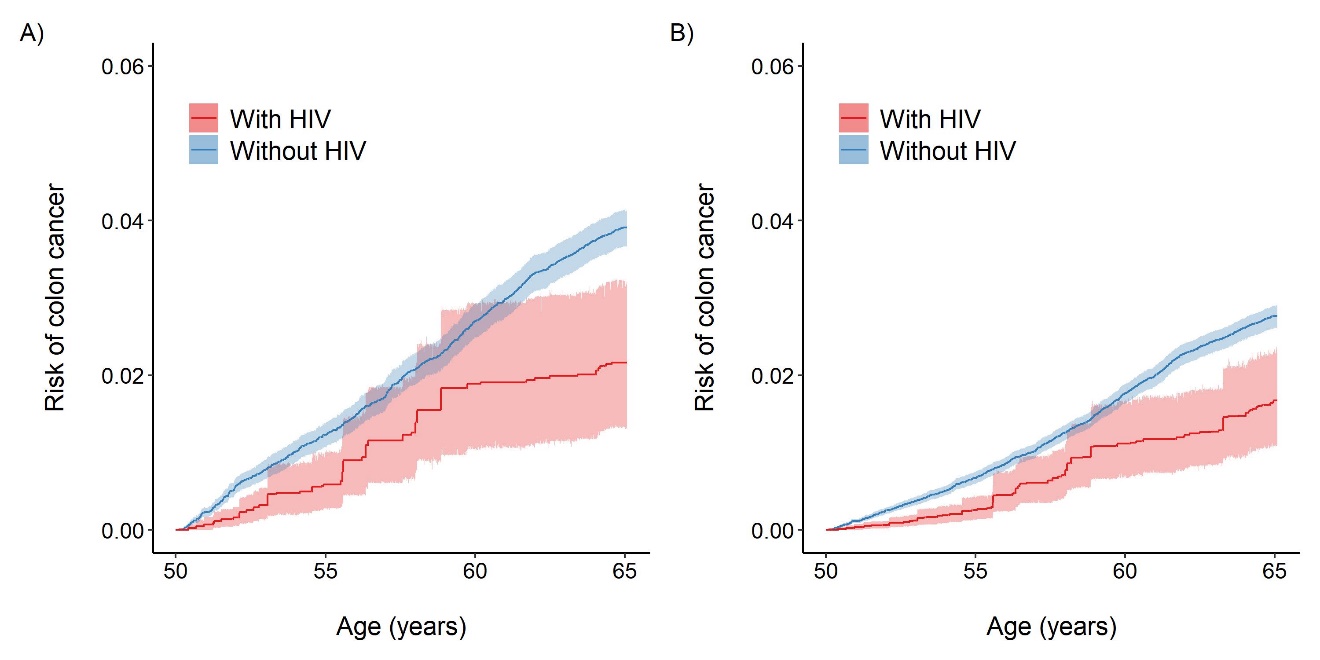


Figure S2. Risk of colon cancer from ages 50 to 64 by HIV status among male beneficiaries, weighting for baseline HIV status and for following screening protocols requiring one endoscopy every (A) two and (B) ten years. Solid lines represent the risk by age and shaded areas represent the 95% confidence intervals for those risks. Blue represents the risk for those without HIV, and red represents the risk for those with HIV.

Table S4. Risk (%) of colon cancer and risk contrasts by HIV status and race/ethnicity, for each screening protocol definition

| Age | 2 Years | | | | 10 Years | | | |
| --- | --- | --- | --- | --- | --- | --- | --- | --- |
|  | With HIV | Without HIV | RD | RR | With HIV | Without HIV | RD | RR |
| *Non-Hispanic Black beneficiaries* | | | | | | | | |
| 55 | 1.0 | 2.1 | -1.1  (-1.7, -0.3) | 0.48  (0.22, 0.85) | 0.5 | 0.9 | -0.5  (-0.7, -0.2) | 0.5  (0.32, 0.77) |
| 60 | 1.6 | 3.7 | -2.1  (-3.0, -1.2) | 0.43  (0.21, 0.68) | 1.2 | 2.2 | -1  (-1.4, -0.5) | 0.55  (0.37, 0.78) |
| 65 | 1.9 | 4.7 | -2.9  (-3.8, -1.9) | 0.40  (0.23, 0.59) | 1.7 | 3.0 | -1.3  (-1.9, -0.8) | 0.57  (0.38, 0.75) |
| *Non-Hispanic white beneficiaries* | | | | | | | | |
| 55 | 0.8 | 1.6 | -0.9  (-1.5, -0.1) | 0.48  (0.13, 0.95) | 0.4 | 0.9 | -0.5  (-0.8, -0.1) | 0.47  (0.16, 0.90) |
| 60 | 1.7 | 3.0 | -1.3  (-2.5, -0.1) | 0.56  (0.18, 0.97) | 1.4 | 2.0 | -0.6  (-1.5, 0.5) | 0.70  (0.25, 1.29) |
| 65 | 1.9 | 3.8 | -1.9  (-3.1, -0.4) | 0.51  (0.20, 0.90) | 1.8 | 2.6 | -0.8  (-1.8, 0.7) | 0.69  (0.31, 1.28) |
| *Hispanic beneficiaries* | | | | | | | | |
| 55 | 0.1 | 1.2 | -1.1  (-1.4, -0.7) | 0.10  (0.00, 0.27) | 0.1 | 0.7 | -0.6  (-0.7, -0.4) | 0.11  (0.00, 0.25) |
| 60 | 0.9 | 2.5 | -1.6  (-2.7, 0.3) | 0.35  (0.00, 1.12) | 0.6 | 1.5 | -1  (-1.5, -0.2) | 0.38  (0.08, 0.90) |
| 65 | 1.2 | 3.2 | -2.0  (-3.4, -0.1) | 0.37  (0.01, 0.97) | 0.9 | 2.1 | -1.1  (-1.9, -0.3) | 0.45  (0.09, 0.83) |


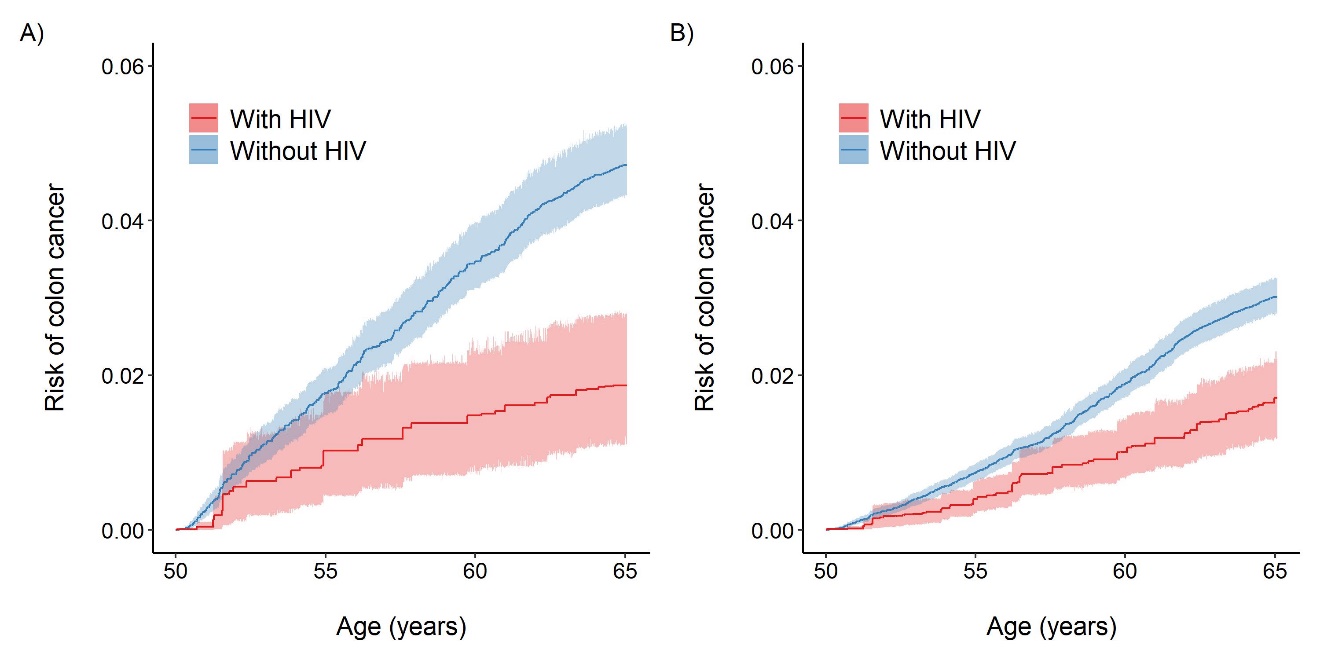


Figure S3. Risk of colon cancer from ages 50 to 64 by HIV status among non-Hispanic Black beneficiaries, weighting for baseline HIV status and for following screening protocols requiring one endoscopy every (A) two and (B) ten years. Solid lines represent the risk by age and shaded areas represent the 95% confidence intervals for those risks. Blue represents the risk for those without HIV, and red represents the risk for those with HIV.


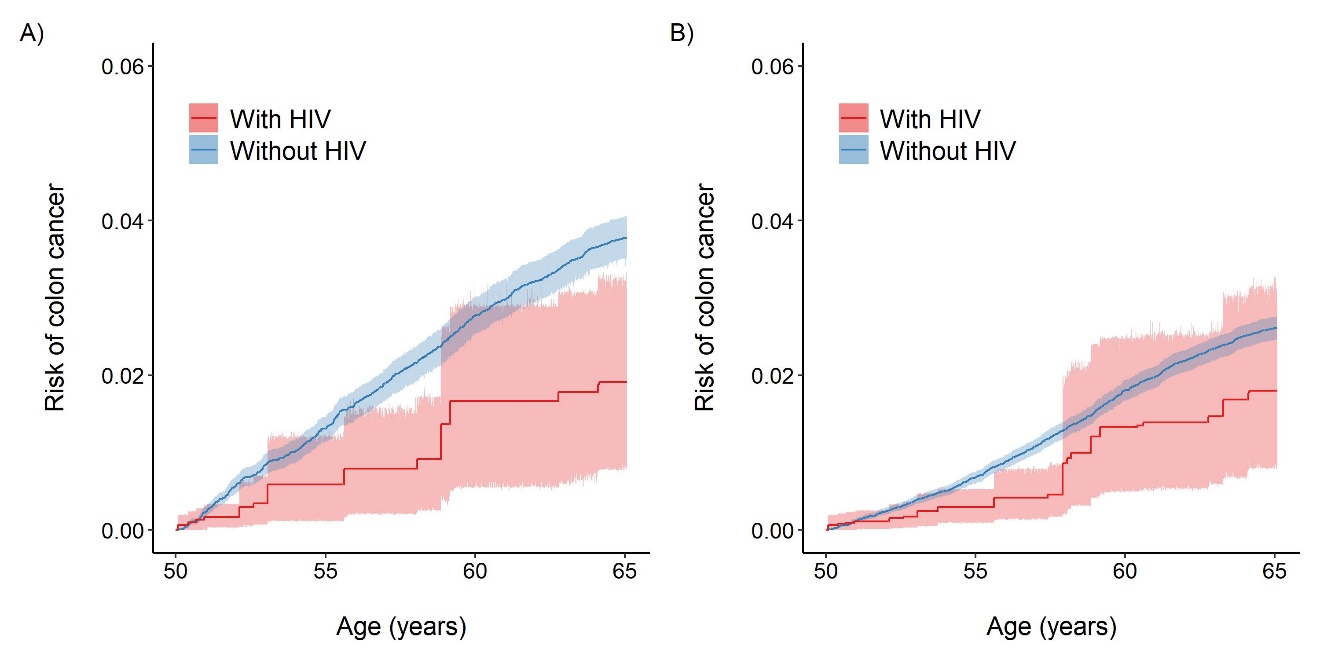


Figure S4. Risk of colon cancer from ages 50 to 64 by HIV status among non-Hispanic white beneficiaries, weighting for baseline HIV status and for following screening protocols requiring one endoscopy every (A) two and (B) ten years. Solid lines represent the risk by age and shaded areas represent the 95% confidence intervals for those risks. Blue represents the risk for those without HIV, and red represents the risk for those with HIV.


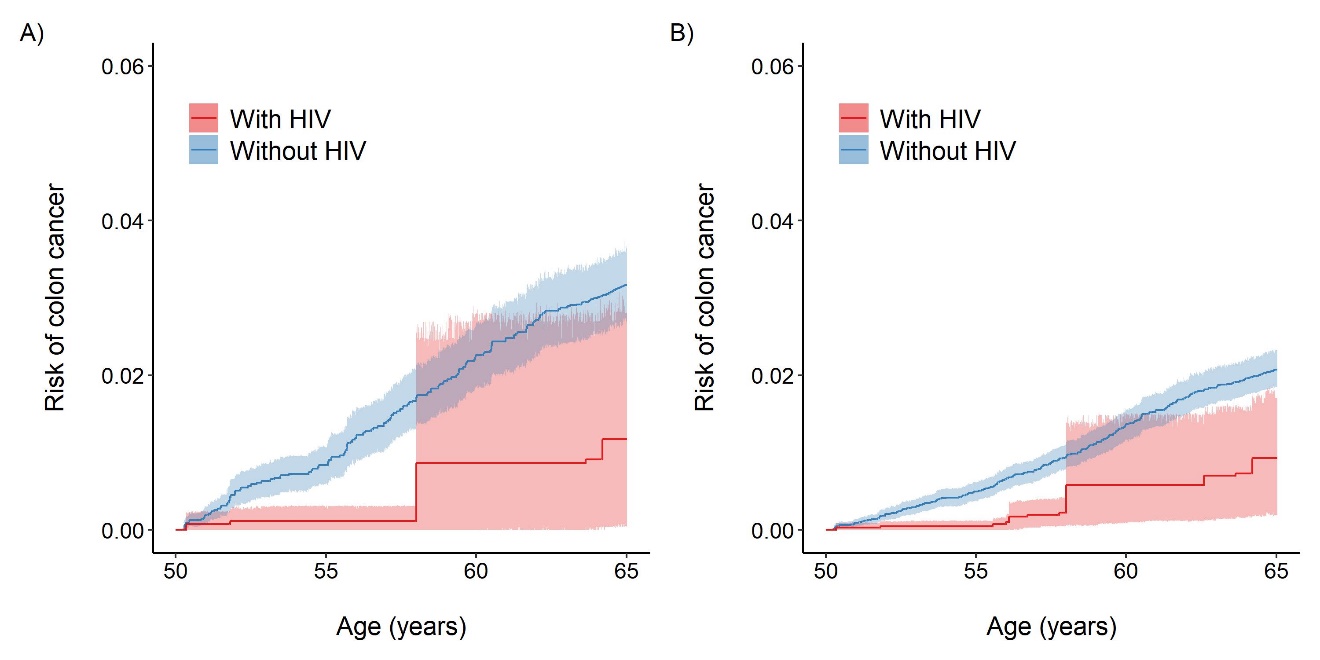


Figure S5. Risk of colon cancer from ages 50 to 64 by HIV status among Hispanic beneficiaries, weighting for baseline HIV status and for following screening protocols requiring one endoscopy every (A) two and (B) ten years. Solid lines represent the risk by age and shaded areas represent the 95% confidence intervals for those risks. Blue represents the risk for those without HIV, and red represents the risk for those with HIV.

Table S5. Risk (%) of death and risk contrasts by HIV status, for each screening protocol definition

| Age | 2 Years | | | | 10 Years | | | | |
| --- | --- | --- | --- | --- | --- | --- | --- | --- | --- |
|  | With HIV | Without HIV | RD | RR | With HIV | Without HIV | RD | RR | |
| *All beneficiaries* | | | | | | | | |  |
| 55 | 37.1 | 13.2 | 23.9  (21.3, 26.7) | 2.81  (2.59, 3.03) | 18.7 | 7.1 | 11.6  (10.4, 12.8) | 2.63  (2.45, 2.81) | |
| 60 | 52.6 | 22.5 | 30.1  (27.5, 33.1) | 2.34  (2.22, 2.48) | 33.5 | 15.8 | 17.7  (15.8, 19.8) | 2.12  (2.00, 2.25) | |
| 65 | 54.8 | 26.2 | 28.7  (26.3, 31.4) | 2.10  (1.99, 2.20) | 38.5 | 20.0 | 18.5  (16.5, 20.5) | 1.93  (1.82, 2.02) | |
| *Female beneficiaries* | | | | | | | | |  |
| 55 | 40.4 | 11.5 | 29.0  (23.7, 34.6) | 3.53  (3.05, 4.05) | 18.9 | 5.9 | 13.0  (10.5, 15.5) | 3.21  (2.78, 3.64) | |
| 60 | 56.1 | 18.8 | 37.3  (31.4, 42.6) | 2.99  (2.64, 3.29) | 35.5 | 13.0 | 22.5  (17.7, 27.7) | 2.74  (2.36, 3.13) | |
| 65 | 57.4 | 21.4 | 36.0  (30.5, 42.0) | 2.68  (2.42, 2.99) | 39.2 | 16.2 | 23.0  (18.3, 27.9) | 2.42  (2.14, 2.72) | |
| *Male beneficiaries* | | | | | | | | |  |
| 55 | 34.4 | 15.3 | 19.1  (16.4, 21.4) | 2.24  (2.06, 2.42) | 18.2 | 8.6 | 9.5  (8.2, 10.6) | 2.10  (1.94, 2.23) | |
| 60 | 49.9 | 26.8 | 23.1  (20.0, 26.1) | 1.87  (1.75, 1.98) | 32.8 | 19.0 | 13.8  (11.9, 15.8) | 1.73  (1.63, 1.83) | |
| 65 | 53.1 | 31.3 | 21.8  (18.4, 24.5) | 1.70  (1.58, 1.78) | 38.3 | 24.2 | 14.1  (12.2, 15.9) | 1.58  (1.50, 1.66) | |

Table S6. Risk (%) of death and risk contrasts by HIV status and race-ethnicity, for each screening protocol definition

| Age | 2 Years | | | | 10 Years | | | | |
| --- | --- | --- | --- | --- | --- | --- | --- | --- | --- |
|  | With HIV | Without HIV | RD | RR | With HIV | Without HIV | RD | RR | |
| *Non-Hispanic Black beneficiaries* | | | | | | | | |  |
| 55 | 52.5 | 20.7 | 31.8  (28.3, 34.9) | 2.54  (2.34, 2.73) | 24.0 | 9.0 | 15.0  (13.4, 16.2) | 2.66  (2.47, 2.83) | |
| 60 | 71.2 | 33.5 | 37.7  (34.5, 40.7) | 2.12  (2.01, 2.24) | 42.2 | 20.2 | 22.0  (19.9, 24.1) | 2.08  (1.98, 2.20) | |
| 65 | 73.4 | 37.8 | 35.7  (32.5, 38.8) | 1.94  (1.86, 2.05) | 48.5 | 25.5 | 23.0  (20.7, 25.5) | 1.90  (1.81, 2.01) | |
| *Non-Hispanic white beneficiaries* | | | | | | | | |  |
| 55 | 41.2 | 16.4 | 24.8  (18.7, 30.6) | 2.51  (2.14, 2.88) | 22.7 | 9.5 | 13.2  (10.5, 16.1) | 2.39  (2.09, 2.69) | |
| 60 | 57.0 | 27.9 | 29.1  (22.9, 34.1) | 2.05  (1.84, 2.24) | 38.8 | 20.0 | 18.8  (14.5, 23.2) | 1.94  (1.73, 2.17) | |
| 65 | 58.8 | 32.3 | 26.5  (20.6, 31.3) | 1.82  (1.64, 1.97) | 42.9 | 25.2 | 17.7  (14.1, 21.6) | 1.70  (1.55, 1.86) | |
| *Hispanic beneficiaries* | | | | | | | | |  |
| 55 | 25.8 | 5.1 | 20.6  (13.1, 26.5) | 5.05  (3.49, 6.36) | 11.9 | 2.8 | 9.1  (5.2, 11.6) | 4.30  (2.82, 5.28) | |
| 60 | 41.0 | 9.1 | 31.9  (21.1, 41.4) | 4.53  (3.27, 5.60) | 22.9 | 6.8 | 16.1  (9.9, 20.7) | 3.38  (2.48, 4.11) | |
| 65 | 42.2 | 10.9 | 31.3  (21.5, 41.4) | 3.87  (2.99, 4.99) | 23.9 | 8.8 | 15.1  (10.2, 19.6) | 2.71  (2.16, 3.25) | |

Table S7. Risk (%) of colon cancer and risk contrasts by HIV status, under sensitivity analyses

| Age | 2 Years | | | | 4 Years | | | | 10 Years | | | |
| --- | --- | --- | --- | --- | --- | --- | --- | --- | --- | --- | --- | --- |
|  | With HIV | Without HIV | RD | RR | With HIV | Without HIV | RD | RR | With HIV | Without HIV | RD | RR |
| *Subset to 2010-2015* | | | | | | | | | | | | |
| 55 | 0.7 | 1.2 | -0.5  (-0.9, 0.2) | 0.61  (0.24, 1.18) | 0.5 | 1.0 | -0.5  (-0.8, -0.1) | 0.49  (0.21, 0.92) | 0.3 | 0.6 | -0.4  (-0.5, -0.1) | 0.45  (0.21, 0.81) |
| 60 | 1.4 | 2.4 | -1.0  (-1.7, -0.1) | 0.59  (0.32, 0.95) | 1.4 | 2.2 | -0.8  (-1.4, -0.1) | 0.62  (0.39, 0.97) | 0.8 | 1.5 | -0.7  (-1.0, -0.2) | 0.54  (0.34, 0.84) |
| 65 | 2.1 | 3.1 | -1.0  (-1.8, 0.0) | 0.68  (0.43, 0.99) | 1.9 | 2.9 | -0.9  (-1.6, -0.1) | 0.68  (0.46, 0.97) | 1.5 | 2.2 | -0.7  (-1.2, -0.2) | 0.67  (0.45, 0.93) |
| *Related endoscopies within 6 months* | | | | | | | | | | | | |
| 55 | 0.9 | 1.7 | -0.7  (-1.2, -0.2) | 0.56  (0.31, 0.87) | 0.8 | 1.6 | -0.8  (-1.1, -0.4) | 0.49  (0.31, 0.72) | 0.5 | 0.9 | -0.4  (-0.6, -0.2) | 0.56  (0.37, 0.82) |
| 60 | 1.7 | 2.9 | -1.3  (-2.0, -0.4) | 0.56  (0.34, 0.85) | 1.7 | 3.0 | -1.4  (-2.0, -0.6) | 0.55  (0.35, 0.80) | 1.2 | 1.9 | -0.7  (-1.1, -0.2) | 0.65  (0.45, 0.88) |
| 65 | 2.1 | 3.8 | -1.6  (-2.4, -0.8) | 0.57  (0.38, 0.80) | 2.2 | 3.7 | -1.5  (-2.2, -0.8) | 0.59  (0.40, 0.79) | 1.8 | 2.6 | -0.8  (-1.3, -0.3) | 0.70  (0.50, 0.89) |
| *Only colonoscopies* | | | | | | | | | | | | |
| 55 | 0.9 | 1.5 | -0.5  (-1.0, 0.1) | 0.65  (0.35, 1.03) | 0.8 | 1.4 | -0.7  (-1.0, -0.3) | 0.53  (0.31, 0.79) | 0.5 | 0.8 | -0.3  (-0.5, -0.1) | 0.63  (0.41, 0.93) |
| 60 | 1.7 | 2.8 | -1.1  (-1.7, -0.3) | 0.60  (0.38, 0.88) | 1.7 | 3.0 | -1.3  (-1.9, -0.5) | 0.56  (0.35, 0.81) | 1.3 | 1.9 | -0.6  (-1.0, -0.2) | 0.67  (0.47, 0.89) |
| 65 | 2.2 | 3.7 | -1.5  (-2.3, -0.8) | 0.58  (0.39, 0.79) | 2.2 | 3.7 | -1.5  (-2.2, -0.8) | 0.59  (0.40, 0.78) | 1.8 | 2.6 | -0.7  (-1.2, -0.2) | 0.71  (0.52, 0.93) |

Table S8. Risk (%) of colon cancer, death, and risk contrasts by HIV status, for the 4-year screening protocol definition

| Age | Colon Cancer | | | | Death | | | |  |
| --- | --- | --- | --- | --- | --- | --- | --- | --- | --- |
|  | With HIV | Without HIV | RD | RR | With HIV | Without HIV | RD | RR | |
| *All beneficiaries* | | | | | | | | |  |
| 55 | 0.8 | 1.6 | -0.8  (-1.1, -0.4) | 0.50  (0.31, 0.72) | 33.5 | 13.2 | 20.3  (18.1, 22.5) | 2.54  (2.37, 2.71) | |
| 60 | 1.7 | 3.0 | -1.3  (-2.0, -0.6) | 0.55  (0.35, 0.80) | 53.3 | 24.7 | 28.6  (26.2, 31.2) | 2.16  (2.06, 2.27) | |
| 65 | 2.2 | 3.7 | -1.5  (-2.2, -0.7) | 0.59  (0.40, 0.80) | 56.3 | 28.2 | 28.1  (25.8, 30.7) | 1.99  (1.91, 2.09) | |
| *Female beneficiaries* | | | | | | | | |  |
| 55 | 0.6 | 1.6 | -1.0  (-1.4, -0.5) | 0.39  (0.19, 0.72) | 37.3 | 11.6 | 25.7  (20.8, 29.8) | 3.22  (2.79, 3.59) | |
| 60 | 1.6 | 3.2 | -1.6  (-2.6, -0.1) | 0.49  (0.20, 0.96) | 56.6 | 21.2 | 35.4  (30.1, 40.2) | 2.67  (2.41, 2.90) | |
| 65 | 2.1 | 3.8 | -1.7  (-2.7, -0.2) | 0.56  (0.31, 0.95) | 59.2 | 23.9 | 35.3  (30.2, 39.8) | 2.48  (2.27, 2.68) | |
| *Male beneficiaries* | | | | | | | | |  |
| 55 | 0.8 | 1.4 | -0.7  (-1.0, -0.1) | 0.54  (0.27, 0.93) | 31.0 | 15.0 | 16.0  (13.6, 17.8) | 2.06  (1.90, 2.18) | |
| 60 | 1.7 | 3.0 | -1.4  (-2.1, -0.5) | 0.55  (0.32, 0.84) | 50.3 | 28.3 | 22.1  (19.2, 24.6) | 1.78  (1.67, 1.87) | |
| 65 | 2.1 | 3.8 | -1.7  (-2.4, -0.6) | 0.55  (0.36, 0.84) | 53.8 | 32.9 | 20.9  (17.8, 23.2) | 1.64  (1.54, 1.71) | |
